# Ontogenetic shift and feeding habits of the European hake (*Merluccius merluccius* L., 1758) in Central and Southern Tyrrhenian Sea (Western Mediterranean Sea): A comparison between past and present data

**DOI:** 10.1002/ece3.8634

**Published:** 2022-03-23

**Authors:** Claudio D’Iglio, Nunziatina Porcino, Serena Savoca, Adriana Profeta, Anna Perdichizzi, Enrico Armeli Minicante, Davide Salvati, Francesco Soraci, Paola Rinelli, Daniela Giordano

**Affiliations:** ^1^ Institute for Marine Biological Resources and Biotechnology (IRBIM) National Research Council Messina Italy; ^2^ Department of Chemical, Biological, Pharmaceutical and Environmental Sciences University of Messina Messina Italy; ^3^ Department of Biomedical, Dental and Morphological and Functional Imaging University of Messina Messina Italy

**Keywords:** Central Mediterranean, energy flow, feeding habits, *Merluccius merluccius*, ontogenetic diet shift

## Abstract

The present paper aims to investigate the ecological role of *Merluccius merluccius*, Linnaeus, 1758, in southern and central Tyrrhenian Sea (GSA 10, Resolution GFCM/33/2009/2 General Fisheries Commission for the Mediterranean), analyzing ontogenetic diet shifts, geographical variations on prey composition, and feeding habits. A total of 734 hake specimens ranging in size between 6 cm and 73 cm (Total Length, TL) were collected in 2018. In order to evaluate ontogenetic shifts in prey composition, samples were divided into five size classes and for each class the quantitative feeding indices have been calculated. The statistical analysis, based on index of relative importance percentage (%IRI), resulted in three trophic groups. The most abundant prey found in the immature hake specimens (size class I) were the Euphausiids, *Stylocheiron longicorne* and Mysidacea, while for samples with a total length over 10.5 cm were crustaceans and fish. *Engraulis encrasicolus* was the most abundant fish prey identified, followed by *Boops boops* and Myctophids. The high presence of Euphausiids, Mysids, Myctophidae, and Sternoptychidae in classes I, II, II, and IV (6–23 cm) showed the relevant role of mesopelagic fauna in hake diets, with an essential organic matter and energy flow from the mesopelagic to the epipelagic environment. Additionally, decapod crustaceans were found in the stomach contents of hakes belonging to class V (with size over 36 cm TL), which is notable considering that our study area includes an important decapod crustacean fishing area.

## Graphical abstract

This study investigates the feeding habits of European hake in the southern Tyrrhenian Sea. It is a clear example of inverse energy flow between mesopelagic and epipelagic biocenosis. The high occurrence of Euphausiids, Myctophids, and Sternoptychids found in gut contents of juvenile hake showed the importance of energy and organic matter from deeper strata for the growth of young hakes.

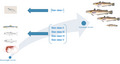

## 1 INTRODUCTION

Mediterranean Sea is one of the most exploited ecosystems in the world, with a very high anthropogenic impact related to fisheries activities. According to Colloca et al. (2013), based on Scientific, Technical, and Economic Committee for Fisheries (STECF) data from 2010 (Carvalho & Doerner, 2014), 84% of all Mediterranean stocks were overexploited, with an increase up to 2014 (88%), declining to 75% in 2018 (FAO, 2020). Overexploited stocks include all existing demersal fish stocks, and seven crustaceans stocks out of nine, while a sustainable exploitation level was only showed by small pelagic stocks (e.g., *Engraulis encrasicolus* and *Sardina pilchardus*). Concerning demersal fish stocks, *Merluccius merluccius*, *Mullus barbatus*, *Mullus surmuletus*, *Pagellus erythrinus*, and *Pagellus acarne* are the most overexploited, especially in Central and Western Mediterranean Sea, with the 15.2% (179,000 t) of total landings from Mediterranean and Black Sea which belong to Central Mediterranean Sea (FAO, 2020). Concerning the Tyrrhenian Sea, it is one of the most exploited areas in the Central Mediterranean Sea, with a high number of operating fishing vessels.

According to FAO (2018), among the demersal teleost's species of Mediterranean and Black Sea, capture production of European hake *M*. *merluccius* was the highest (20170 t/year) with a continuous decline in catch since the 1980s–1990s, followed by *Boops boops*, Linnaeus, 1758, (19,711 t/year), *M*. *barbatus* (16,092 t/year), and *P*. *erythrinus* (11,658 t/year). Recently, in the GSA10 (Southern Tyrrhenian Sea), despite the decreasing trend of the exploitation rate shown by the recent data of the General Fisheries Commission for the Mediterranean (GFCM), no increase in biomass was recorded, with a delay in the stock response (FAO, 2020). Indeed, the Southern Tyrrhenian Sea is an heavily exploited area, with a large trawling fleet composed by 2800 vessels (the third one for vessels number among the Italian fleets), contributing to 12% of total national landings (IREPA, 2011), and a peculiar heterogeneity of fisheries activities, with an high developed artisanal fisheries characterized by a large variability of fishing methods and target species (Cataudella & Spagnolo, 2011; Giordano, 1999; Perdichizzi et al., 2022) and the presence of one fishery exclusion zone which has been subject to a trawling ban since 1990 (Mangano et al., 2015; Pipitone et al., 2000; Rinelli et al., 2004). Therefore, it is important to monitor commercial fishing and conservation status of the mainly exploited stocks, such as *M*. *merluccius*.

It is essential to fully understand the ecology of several species, especially the most over exploited ones, to improve the multispecies approach of ecosystem‐based fishery management and deepen the knowledge on ecological relationships within the marine trophic chains (D’Iglio, Albano, Tiralongo, et al., 2021; D’Iglio et al., 2022; Möllmann et al., 2014; Pikitch et al., 2004; Savoca et al., 2020; Stagioni et al., 2011; Zhou et al., 2010). Trophic network and ecological dynamics involving target species in the marine ecosystem can be clarified by a careful study and description of their diet and feeding habits (Angelini et al., 2016; Chipps & Garvey, 2006; Punt et al., 2016; Riccioni et al., 2018). These studies should be increased to prevent a fishing‐induced decline of marine food web trophic levels (Shackell et al., 2010) setting also a conscious exploitation across the trophic levels (Garcia, Garcia, et al., 2014; Garcia, Rice, et al., 2014). Prey–predator relationships, and their changes in time, are the basis of multispecies population dynamics and complex ecosystem models (Carrozzi et al., 2019). The abundance decrease of piscivorous predators in Mediterranean Sea related to fisheries overexploitation (Colloca et al., 2013) is a serious problem for marine ecosystems, with poorly understood consequences for entire marine food chains (Leroux & Loreau, 2015; Lotze et al., 2011).

The European hake (*Merluccius merluccius*, Linnaeus, 1758) is an essential predator of the deeper shelf–upper slope Mediterranean communities. This species inhabits a wide depth range (20–1000 m) throughout the Mediterranean Sea and the north‐eastern Atlantic regions (Carpentieri et al., 2005; Fischer, 1987) and it is considered a nektobenthic species. A size‐based bathymetric segregation can usually be observed in hake populations, with larger individuals living in waters deeper than 200 m, while juveniles mainly inhabit the coastal shelf (Meiners, 2007). The European hake is an opportunistic predator since its feeding habits show geographical differences in prey richness and availability (Bozzano et al., 2005; Cartes, Hidalgo, et al., 2009; Cartes, Maynou, et al., 2009; Carrozzi et al., 2019; Papaconstantinou & Stergiou, 1995; Velasco & Olaso, 1998). Maybe, this great variety of feeding habits is due to its wide bathymetric distribution (Carpentieri et al., 2005; Cartes, Hidalgo, et al., 2009; Cartes, Maynou, et al., 2009; Cartes et al., 2004). Its opportunistic feeding behavior could also induce specimens to feed on trawl gears discard, represented by organisms killed or injured by this fishing activity, as reported by previous studies on other demersal predator species (D’Iglio, Albano, Famulari, et al., 2021; D’Iglio, Savoca, et al., 2021; Kaiser & Spencer, 1994).

The *M*. *merluccius* specimens show changes in diet composition and feeding habits related to their ontogenetic development. Planktonic species, such as euphausiids and mysids, are mainly preyed by immature and juvenile individuals (total length >15 cm, indicated as size classes I and II in the present paper). These follow the preys (mainly zooplanktonic species as Euphausiacea) during their nictemeral vertical migration, moving upward and downward through the water column (Orsi Relini et al., 1997). Small fishes, cephalopods, and nektobenthic decapods are mainly preyed by hake individuals with a total length from 10.5 to 20 cm, (indicated as size classes II and III in the present paper). Finally, larger pelagic and nektobenthic fishes are the typical preys of mature hakes, up to 20 cm of total length (size classes IV and V of present paper).

Although many authors deal with this topic through all the Mediterranean areas, there are only two studies focused on European hake diet in the Central and Southern Tyrrhenian (Modica et al., 2015; Sinopoli, 2012).

The aim of the present study was to investigate, in relation to the size class, the changes in the diet composition and in the feeding habits of the species *Merluccius merluccius* from the central and southern Tyrrhenian Sea. Monitoring trophic variations in overexploited species of high commercial value is essential for stocks and demersal community conservation, improving fisheries management, especially in a high exploited area, such as the Southern Tyrrhenian Sea.

## 2 MATERIALS AND METHODS

### 2.1 Study area

**FIGURE 1:**
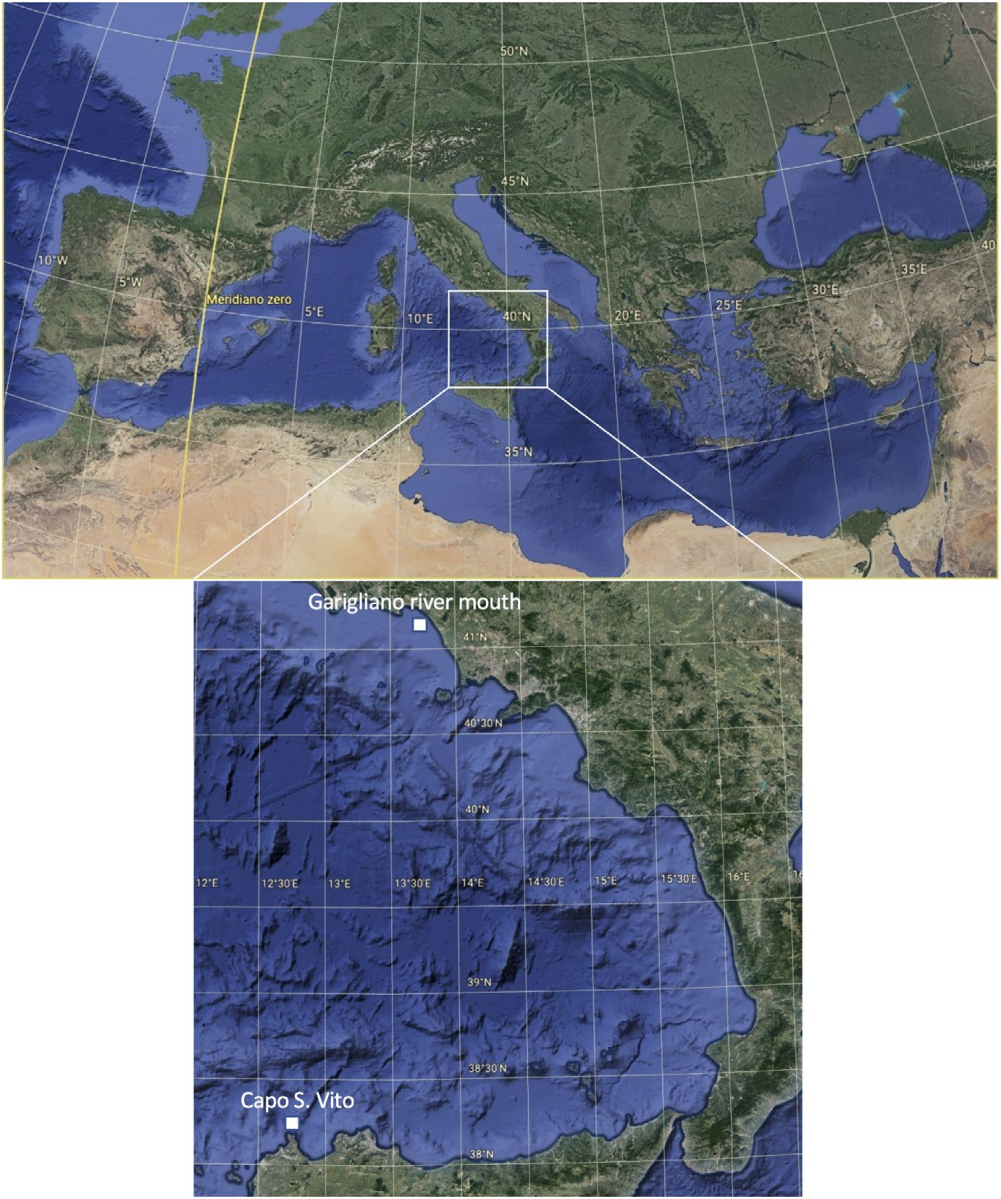
Sampling area (GSA10) from Garigliano river to San Vito Cape

The investigated area extends in southern and central Tyrrhenian Sea (GSA10) (Figure 1), from the Garigliano river mouth, on the Lazio border, to Capo S. Vito, the westernmost part of northern Sicily, covering a 20,225 km^2^ area, with a bathymetric depth between 10 and 800 m. Its coastal extension is 1129 km, involving five regions: Lazio, Basilicata, Campania, Calabria, and Sicily. GSA10 is heavily exploited by a large trawling fleet. Moreover, it is characterized by one fishery exclusion zone which has been subject to a trawling ban since 1990 (Pipitone et al., 2000; Rinelli et al., 2004) and a region typically oligotrophic, in the southern part (Povero et al., 1990).

Furthermore, the Tyrrhenian coasts are among the most polluted ones of Mediterranean area, like eastern Campania (“Land of Fire”) and many others in the entire area (Tamburrino et al., 2019; Triassi et al., 2019; Vichi et al., 2019). Decades of waste dumping practices, associated with the widespread use of pesticides and fertilizers, incineration processes, and massive release of environmental contaminants, have contaminated the whole environment, including the marine coastal ecosystem (Ariano et al., 2019; Esposito et al., 2018; Legambiente, 2003, 2007, 2018; Piazzese et al., 2019; Rodhouse et al., 2015; Senior & Mazza, 2004).

### 2.2 Stomach data

A total of 734 European hakes, *M*. *merluccius*, individuals ranging from 6 and 73 cm total length (TL) were collected from the GSA10 during Summer (by MEDITS 2018 bottom‐trawl survey) and during the entire year by commercial landings of the fishing fleets (CAMPBIOL 2018 survey). The collected samples were frozen on board to prevent digestion of stomach contents and taken to the laboratory, where each specimen was measured (total length, TL cm), weighed (total weight, TW g), and sexed with the relative degree of sexual maturity (Follesa & Carbonara, 2019).

Stomach repletion status was detected using a five‐stage macroscopic scale (1 empty; 2 full <50%; 3 full >50%; 4 bursting; 5 everted, stomach inside the mouth). The stomachs were preserved in ethanol 70% + glycerin 5%. Each prey item was identified to the lowest possible taxonomic level, counted and weighed; for each one (species or major Taxa) was detected the digestion degree (1 = undamaged; 2 = almost digested; 3 = highly digested). The anatomical undigested parts, as mouth parts, entire heads capsules, otoliths, eyes, telsons, carapace, were used to count any prey. The double‐counting was avoided by considering only one kind of anatomical remains for each preys group, depending on their presence within the stomach contents (e.g., only carapace or telsons, only eyes or otoliths, only heads capsules or fishes’ columns).

Prey items in a state of advanced digestion were grouped into undetermined fish, crustaceans, and cephalopods. The vacuity index (VC), obtained by VC = (*N_e_*/*N*) × 100, where Ne is the number of empty stomachs and *N* is the number of total stomachs, was calculated; in the VC we did not consider the everted stomachs. To evaluate the contribution of each prey items, the following parameters were calculated: the percentage of abundance composition (%*N*), the percentage of biomass composition (%W), and the frequency of occurrence (%*F*) (Hyslop, 1980). These indices are necessary to calculate the Relative Importance Index, IRI = %*F* (%*N* + %*P*), expressed as $\%\text{IRI}=\left({\text{IRI}}_{i}\sum_{i}^{N}{\text{IRI}}_{i}\right)$ x 100 (Cortés, 1997). To evaluate the diet variations related to growth, five‐length classes were assessed using an incremental scale by 5 cm for length classes I, II, and III. The first class (I) included all immature specimens (<10 cm). The second class (II) included specimens with a total length between 10.5 and 15 cm, while to the third class (III) belonged individuals with a total length between 15.5 and 20 cm. The largest individuals (>20.5 cm) were grouped into two heterogeneous classes: IV (20.5–32.5 cm of total length) and V (total length >32.5 cm).

### 2.3 Statistical analysis

Information on dietary composition was extracted through a combinate univariate and multivariate data analysis. One‐way ANOVA analysis followed by Tukey's test was performed to assess dietary composition differences between the maturity stage of the *M*. *merluccius* specimens. A square root transformation was applied to the dietary composition matrix, then the Bray–Curtis similarities were calculated. The dendrogram was created by means of the average linkage clustering method. Non‐parametric multidimensional scaling (nMDS) ordination was applied to the dietary composition matrix in order to observe the effect of maturity stage on dietary composition. A principal coordinates analysis (PCoA) was used to visualize diet data.

Univariate and multivariate statistical analyses were performed by using Past (V. 4) and PRIMER6‐E. *p* value was set at *p* < .05.

## 3 RESULTS

As shown in Table 1, the vacuity index (VC) was increased in value from size classes I to II, decreasing in the other size classes. Concerning the everted stomachs number, size class IV showed the highest value, followed by size class III, with the lowest value showed by size class V.

**TABLE 1:** Number of sampled *Merluccius merluccius* individuals with the number of stomachs sampled and analyzed in the southern and central Tyrrhenian Sea (GSA 10) by size classes. The vacuity index (%VC) and the number of Empty, Full <50%, Full >50%, Bursting and Everted stomachs are also shown

Size class	Total length	Empty	Full <50%	Full >50%	Bursting	Everted	Individuals Sampled	Stomachs Sampled	Stomachs Analyzed^a^	VC = (*N_e_*/*N*)*100
I	<10 cm LT	42	4	5	14	41	106	23	20	39.6
II	10.5–15 cm LT	92	14	16	33	76	231	60	32	39.8
III	15.5–20 cm LT	31	13	10	26	35	115	50	29	26.9
IV	20.5–32.5 cm LT	44	20	29	56	59	208	110	50	21.1
V	>32.5 cm LT	20	10	7	13	24	74	31	18	27
Ʃ		229	61	67	142	235	734	274	149	31,2

^a^ Stomachs analyzed for taxonomic evaluation.

The content analysis of 274 sampled stomachs showed a total of 120 preys, which were found to belong to 28 taxa. The overall analysis of the diet, based on the %IRI, showed that fishes were the preferred prey (% IRI = 95.29; %*F* = 80.5), followed by Mysids (%IRI = 2.57) and Euphausiids (% IRI = 1.25) (Table 2).

**TABLE 2:** Diet composition of the *Merluccius merluccius* specimens collected from southern and central Tyrrhenian Sea (GSA 10) with %F (frequency of occurrence), %W (percentage in biomass), %N (percentage of number) IRI (index of relative importance) and %IRI (index of relative importance expressed as percentage) values for each prey item

Taxa	%F	%W	%N	IRI	%IRI
*Solenocera membranacea*	0.67	0.15	0.31	0.31	0.003
*Processa acutirostris*	2.01	0.23	0.93	2.32	0.020
*Plesionika martia*	0.67	0.28	0.31	0.39	0.003
*Aristaeomorpha foliacea*	1.34	2.96	0.62	4.80	0.040
*Pasiphae sivado*	0.67	0.08	0.31	0.26	0.002
*Pseudosquillopsis cerisii*	0.67	0.40	0.31	0.47	0.004
*Pontocaris cataphracta*	0.67	0.21	0.31	0.35	0.003
Decapoda	2.68	0.25	1.23	3.97	0.033
Total Decapoda	9.40	4.55	4.32	83.40	0.701
Meganyctiphanes norvegica	1.34	0.34	0.62	1.29	0.011
Stylocheiron sp.	2.68	0.12	1.23	3.65	0.031
Euphausiacea	10.07	0.20	4.63	48.58	0.408
Total Euphausiacea	53.69	0.32	24.69	1342.87	11.283
*Mysidacea* nd.	20.81	0.06	9.57	200.21	1.682
Total Mysidacea	20.81	0.06	9.57	200.21	1.682
*Crustacea*	3.36	0.22	1.54	5.92	0.050
*Rondeletiola minor*	0.67	0.21	0.31	0.35	0.003
*Cephalopoda*	1.34	0.01	0.62	0.84	0.007
Total Cephalopoda	2.68	0.22	1.23	3.91	0.033
*Boops boops*	3.36	20.96	1.54	75.52	0.635
*Chlorophthalmus agassizi*	1.34	6.09	0.62	9.01	0.076
*Capros aper*	0.67	0.12	0.31	0.29	0.002
*Cepola macrophtalma*	2.01	1.18	0.93	4.24	0.036
*Ceratoscopelus maderensis*	2.01	0.43	0.93	2.73	0.023
*Conger conger*	0.67	0.13	0.31	0.29	0.002
*Engraulis encrasicolus*	9.40	22.34	4.32	250.47	2.105
*Hymenocephalus italicus*	1.34	0.24	0.62	1.15	0.010
*Lampanyctus crocodilus*	0.67	0.57	0.31	0.59	0.005
*Trachurus trachurus*	1.34	3.93	0.62	6.10	0.051
*Macroramphosus scolopax*	0.67	0.36	0.31	0.45	0.004
*Maurolicus Muelleri*	0.67	0.16	0.31	0.31	0.003
*Lampanictus crocodilus*	0.67	0.57	0.31	0.59	0.005
Clupeidae	8.05	12.42	3.70	129.88	1.091
Osteichthyes	42.28	17.49	19.44	1561.77	13.122
Ophichthidae	0.67	0.08	0.31	0.26	0.002
Myctophidae	4.70	1.08	2.16	15.20	0.128
*Sparidae Spp*	0.67	1.00	0.31	0.88	0.007
Total Osteichthyes	81.21	89.13	37.35	10271.18	86.301

Note

Frequency of occurrence (%*F*); Percentage in weight (%*W*); Percentage in number (%*N*); Index of relative importance IRI and %IRI.

The Euphausiids and Mysidacea were the most relevant (higher IRI values) prey for immature hake (size class I < 10 cm TL), while decapoda and fish were the preferred prey for hake larger than 10.5 cm of TL (size classes II, III, IV, and V) (Table 3). Among teleosts, *Engraulis encrasicolus* (Linnaeus, 1758) was the most important prey for hake, followed by *Boops boops* (Linnaeus, 1758) and Myctophid (Table 2). Among decapod crustaceans, *Aristaeomorpha foliacea*, (Risso 1827), was the species with the highest IRI value, followed by *Processa acutirostris* (Nouvel & Holthuis, 1957) and *Pseudosquillopsis cerisii* (Roux 1828). Concerning the frequency of occurrence, except for *A*. *foliacea* and *P*. *acutirostris*, most species showed the same %F value (*Solenocera membranacea*, Risso, 1816, *Plesionika martia*, A. Milne‐Edwards 1883, *Pasiphaea sivado*, Risso 1816) (Table 2).

**TABLE 3:** Diet composition of the *Merluccius merluccius* specimens collected from the southern and central Tyrrhenian Sea (GSA 10) in terms of %*F*; %*W*; %*N*; and %IRI relative to each main prey taxa of the five hakes size classes

Taxa	I	II	III	IV	V
*F*%					
Cephalopoda	8.82	0.00	0.00	1.92	0.00
Mysidacea	23.53	0.00	0.00	0.00	0.00
Euphausiacea	26.47	13.79	0.00	0.00	0.00
Decapoda	0.00	0.00	8.00	7.69	21.05
Crustacea n.d.	23.53	3.45	4.00	5.77	5.26
Osteichthyes	17.65	82.76	88.00	84.62	73.68
*N*%					
Cephalopoda	2.25	0.00	0.00	1.05	0.00
Mysidacea	20.79	0.00	0.00	0.00	0.00
Euphausiacea	42.70	12.50	0.00	0.00	0.00
Decapoda	0.00	0.00	20.83	5.26	11.43
Crustacea n.d.	29.78	6.25	4.17	4.21	2.86
Osteichthyes	4.49	81.25	75.00	89.47	85.71
*W*%					
Cephalopoda	1.20	0.00	0.00	0.40	0.00
Mysidacea	8.03	0.00	0.00	0.00	0.00
Euphausiacea	40.16	1.83	0.00	0.00	0.00
Decapoda	0.00	0.00	3.78	0.27	9.81
Crustacea n.d.	17.27	4.06	0.83	0.03	0.27
Osteichthyes	16.67	94.11	95.39	99.30	89.92
IRI%					
Cephalopoda	0.70	0.00	0.00	0.02	0.00
Mysidacea	15.47	0.00	0.00	0.00	0.00
Euphausiacea	50.05	1.34	0.00	0.00	0.00
Decapoda	0.00	0.00	1.29	0.27	3.34
Crustacea n.d.	25.26	0.24	0.13	0.15	0.12
Osteichthyes	8.52	98.42	98.57	99.56	96.54

Regarding Cephalopods, the preys with the highest IRI were unidentified as cephalopod paralarvae and *Rondeletiola minor* (Naef, 1912); while among Osteichthyes, results showed that mesopelagic (Myctophidae n.d., *Lampanyctus crocodilus*, Risso 1810, *Ceratoscopelus maderensis*, Lowe 1839, *Maurolicus muelleri*, Gmelin, 1789), demersal, and pelagic species (*Cepola macrophthalma*, Linnaeus, 1758, *B*. *boops*, *E*. *encrasicolus*, *Trachurus trachurus*, Linnaeus, 1758, *Macroramphosus scolopax*, Linnaeus, 1758) were the most relevant (higher %IRI) (Table 2) and abundant preys (higher %*N*) (Table 2).

**FIGURE 2:**
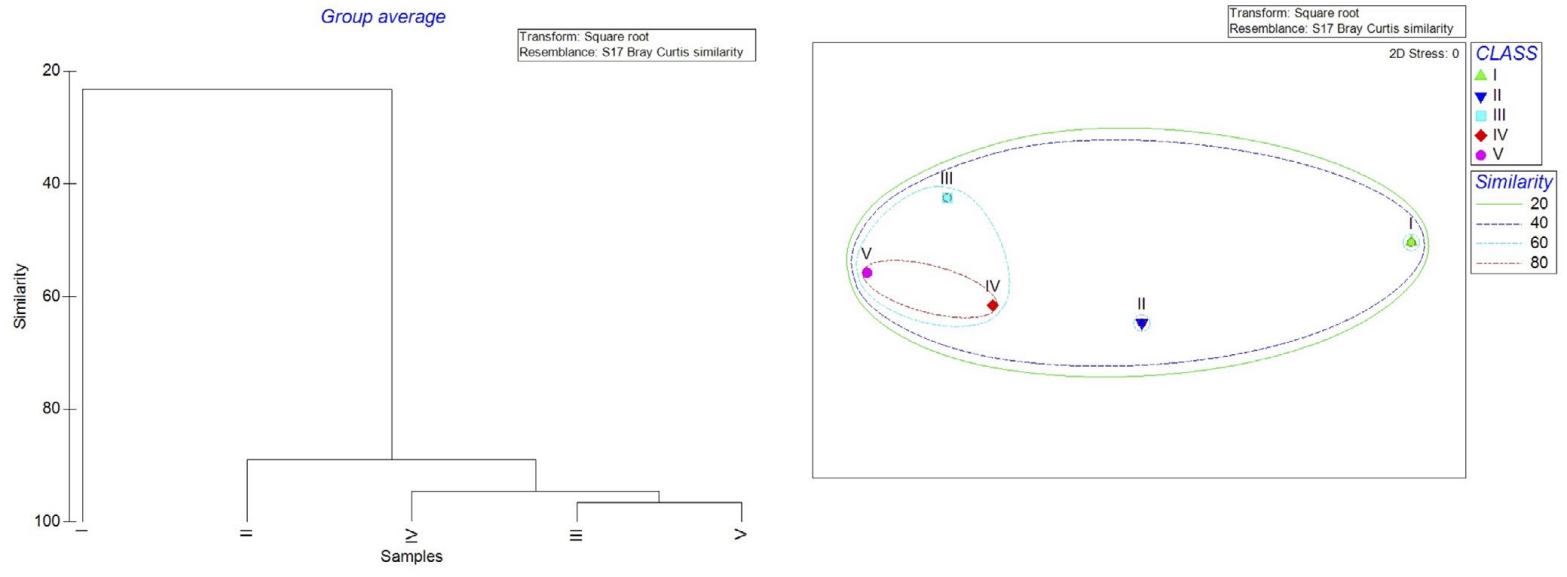
Dendrogram and MDS ordination of Bray–Curtis similarities from dietary data (square root transformation) for the five maturity stages analyzed

Cluster analysis and MDS ordination grouped the whole set of data, by maturity classes, into two main clusters. Cluster I included only the specimens belonging to Class I, while the second one included the specimens belonging to the Classes II, III, IV, and V with similar values of %IRI (Figure 2). In detail, classes III, IV, and V showed the 60% of similarity.

One‐way ANOVA analysis was performed to assess dietary composition differences between maturity stage of the *M*. *merluccius* specimens (*p* < .05). Indeed, class I was different from all other classes (*p* < .05), while classes II, III, IV, and V did not differ significantly in their diets (*p* > .05).

**FIGURE 3:**
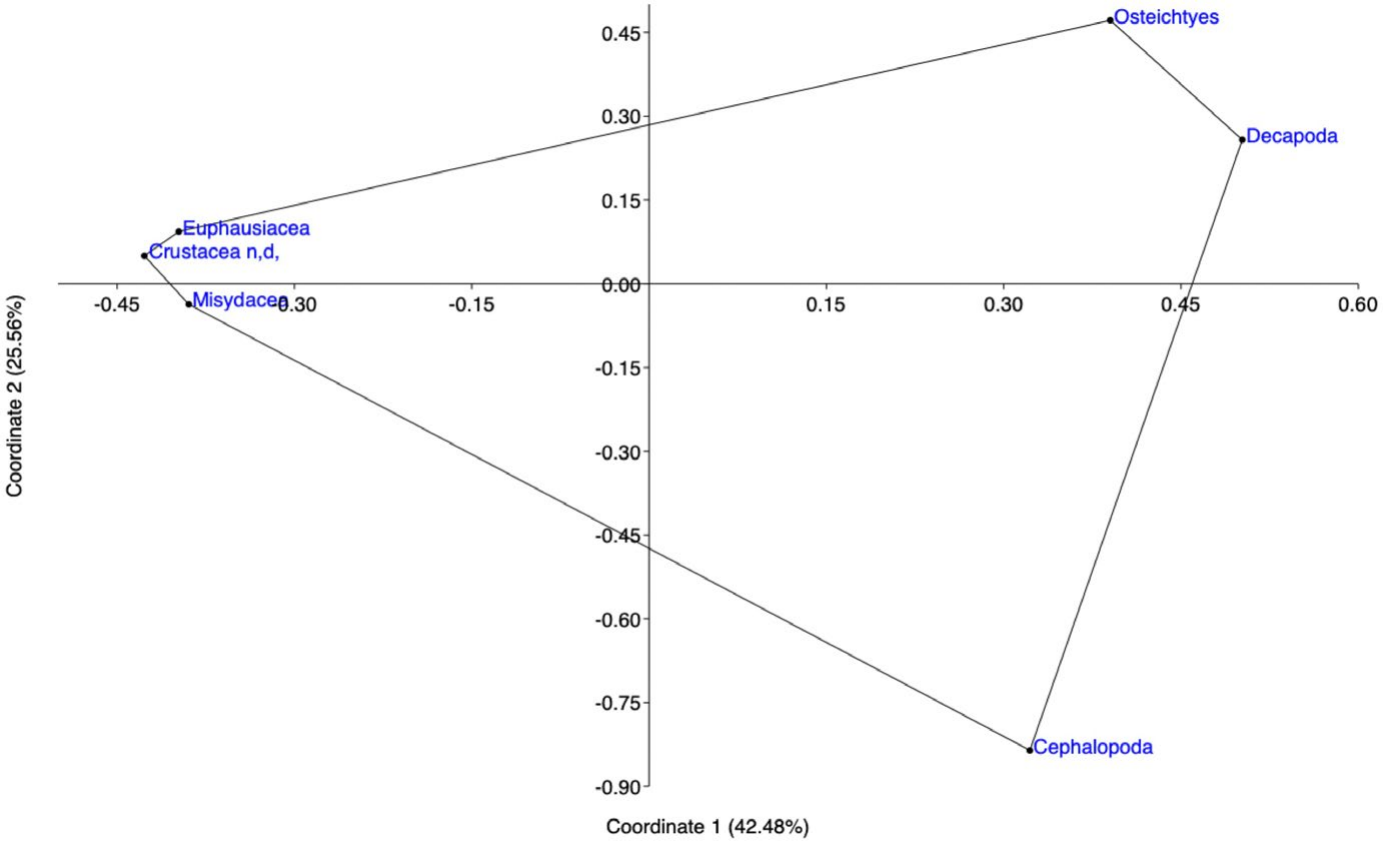
Principal Coordinates Analysis (PCoA) of the stomach contents of the *Merluccius merluccius* specimens based on the Bray–Curtis similarity, which indicates primary prey consumed by each maturity stage

The ANOVA confirmed the similarity of diet between maturity stages, which was driven by the large contributions of Euphausiacea and Osteichthyes as shown by PCoA, explaining 42% of total variation on the first axis (Figure 3).

## 4 DISCUSSION

The present paper provides, for the first time, an analysis of the diet and feeding habits of the *Merluccius merluccius* specimens from the entire area of the central and southern Tyrrhenian Sea (GSA10). Our findings enrich and implement the data already reported by Modica et al. (2015) and Sinopoli (2012), for the southern part of Tyrrhenian (Calabria and Sicily coast). Modica et al. (2015) focused their attention on juvenile hake and their predation on Myctophidae and Sternoptychidae, investigating the area extended from Cape Suvero (western Calabria) to Cape Saint Vito (northern Sicily). While Sinopoli (2012) assessed the trawling effects on *M*. *merluccius* diet and trophodynamic, comparing stomach contents and stable isotopes in samples from three gulfs of northern Sicily. In accordance with the data obtained by Sinopoli (2012) and other authors from different Mediterranean areas (Bozzano et al., 2005; Carpentieri et al., 2005; Cartes, Hidalgo, et al., 2009; Cartes, Maynou, et al., 2009; Cartes et al., 2004), our data confirmed that *M*. *merluccius* is an active predator with a mixed diet based on demersal‐pelagic and benthic preys. It feeds on motile preys (crustaceans or fishes), showing ontogenetic changes on diet with consequent trophic level differentiations among different size classes (Fanelli et al., 2018; Sinopoli, 2012).

European hake diet shifted from Euphausiids (*Meganyctiphanes norvegica*, M. Sars, 1857, *S*. *longicorne*) and Mysids, for immature and juvenile hakes, to fishes and crustacea (mainly bigger Decapoda) for the larger and mature one. Before the transition to a complete ichthyophagous phase, the hakes showed a generalized feeding habit, with a diet dominated by mesopelagic (*C*. *maderensis*, *M*. *muelleri*, *L*. *crocodilus*) and nektonic fish (*E*. *encrasicolus*, *B*. *boops*, *C*. *conger*, ecc.), and characterized by a low incidence of cephalopods. Size‐related dissimilarities in diet composition could be related to genetic needs and/or differences in spatial distribution (Flamigni, 1983; Jukic & Arneri, 1984; Velasco & Olaso, 1998).

As highlighted by Modica et al. (2015), the mesopelagic fauna plays a relevant role in the diet of *M*. *merluccius* from the Tyrrhenian Sea, especially in the southern zone. This is due to the geomorphological features of the area, characterized by abrupt shelf‐slope breaks and steps slopes, such as Balearic Island and Northern side of Mallorca (Carpentieri et al., 2005; Cartes, Hidalgo, et al., 2009; Cartes, Maynou, et al., 2009; Modica et al., 2015). In this kind of habitats, predation on mesopelagic species is most enhanced than in areas with a wide continental shelf, like the central Tyrrhenian and Gulf of Lyon (Bozzano et al., 1997; Ferraton et al., 2007). The geomorphological features of the southern Tyrrhenian, together with the water mass circulation and upwelling phenomena (Gasparini et al., 2005; Marullo et al., 2011; Sparnocchia et al., 1999), promote and increase the vertical migration of mesopelagic fishes toward shallow waters, enhancing the contact between this community and demersal predators, such as *M*. *merluccius*. Moreover, shelf breaks are the hot spots for nursery's formation in the entire Mediterranean Sea. The high density of juveniles belonging to different species, included European hake, is sustained by these habitats, thanks to zooplankton and micronekton aggregations enhanced by primary production increase and convergence processes (Cartes, Hidalgo, et al., 2009; Cartes, Maynou, et al., 2009; Colloca et al., 2004; Fiorentino et al., 2003; Garofalo et al., 2011).

As also shown by the results obtained in this study, the presence of *C*. *maderensis*, together with Euphausiids and Mysids, in the gut contents of hake belonging to different size classes, highlighted the importance of organic matter and energy flow from mesopelagic to epipelagic environment. Moreover, this emphasizes the essential role played by bioluminescent bathypelagic species for the survival and growth of Mediterranean hake.

This energy conveyance by daily migrations of zooplankton and mesopelagic fishes (*C*. *maderensis*, *L*. *crocodilus*, *M*. *muelleri)* seem to be essential for immature (Class I), juvenile (Classes II and III), and large/mature adults’ (Classes IV and V) hakes. Hakes probably prey mesopelagic species (most of them belong to “Deep Scattering Layer”) during their nictemeral migration toward superficial and shallower waters. This is a clear example of inverse energy flow from deeper to epipelagic waters, essential for all demersal species. Since Myctophidae and Sternoptychidae live in depths greater than 600 m (D’Onghia et al., 2004) and hake, especially juveniles and small adults, are generally distributed above 330 m in the studied area (Biagi et al., 2002), we could assert that this area is characterized by an inverse energy flow from deeper to epipelagic water, probably reallocated along the neritic food chain. The upward transfers may be increased by the consumption of mysids and euphausiids in winter–spring and by simultaneous consumption of other fish (e.g., Clupeidae, Argentinidae, Engraulidae, *Chlorophthalmus agassizi*, Bonaparte, 1840, ecc.) that may aggregate in search of the same prey.

It is interesting to focus on the low abundance of Cephalopods in the diet composition of hake from GSA 10. Probably, this trend is due to the ecological and oceanographical features of the investigated area in which the most relevant preys were *E*. *encrasicolus*, *B*. *boop* , and Clupeidae family, in hake belonging to size classes III, IV, and V. In other Mediterranean subarea, such as the Strait of Sicily, these species are mainly preyed by smaller juvenile hakes (Carrozzi et al., 2019; Fanelli et al., 2018), with *E*. *encrasicolus* as the most abundant prey for hake below 14 cm of TL (Classes I and II of present paper), and larger hake which mainly prey on larger bony fishes (*T*. *trachurus*, *Lepidopus caudatus*, Euphrasen, 1788*)* and decapods (Carrozzi et al., 2019). The abundance of preys belonging to the Engraulidae and Clupeidae families in stomach contents of the hake analyzed in the present paper is probably due to the ecological features of the Tyrrhenian Sea, especially in the central region (Bauchot, 1987). In this area, especially during winter and autumn, they are the most available and abundant preys, largely distributed on continental coastal shelf and forming schools usually deeper than 25 m.

This feeding habit highlights and confirms the opportunistic ecological behavior and the generalize niche of nektobenthic predator occupied by European hake in demersal and meso‐ epi‐ pelagic food chain, with trophic level variations during its ontogenetic development (Alheit & Pitcher, 1995; Carpentieri et al., 2005; Cartes, Hidalgo, et al., 2009; Cartes, Maynou, et al., 2009; Mellon‐Duval, 2017; Sinopoli, 2012). This predator, mainly in the adult stage, can increase its prey spectrum, diversifying its feeding habits. *M*. *merluccius* can prey on benthic, supra benthic, and pelagic domain, performing daily vertical migration and oblique displacements (Carpentieri et al., 2005), following the seasonal and daily variations in the distribution and availability of necto‐benthic, benthopelagic, benthic, and pelagic prey categories (Cartes, Hidalgo, et al., 2009; Cartes, Maynou, et al., 2009; Cartes et al., 2004; Papaconstantinou & Stergiou, 1995; Velasco & Olaso, 1998).

Ontogenetic shift in hakes’ diet could be related to the size‐depth distribution of individuals. Immature and juveniles (belonging to size classes I and II) live mostly between 100 and 200 m depth; intermediate hakes (size classes III) reach the highest abundance mainly on the shelf (<100 m); while large hakes (Classes IV and V) live in a wide depth range, concentrate on the shelf break during the spawning period (Alvarez et al., 2001; Colloca et al., 2000; Recasens et al., 1998). Diet composition and trophic habits of hake described by the results reported in this study, such as the ontogenetic changes on the diet, confirmed the high impact of this species on entire demersal communities, including anchovy and other species, as shown by the literature from the Adriatic Sea (Froglia, 1973; Piccinetti & Piccinetti Manfrin, 1971; Riccioni et al., 2018; Zupanovic, 1968) and the Tyrrhenian Sea (Carpentieri et al., 2005).

Further analyses are required to combine the traditional stomach content analysis with the relatively recent techniques of stable isotope analysis and metabarcoding method based on COI PCR amplification. These innovative techniques have been applied to hake trophic ecology in other Mediterranean geographical areas, such as the Adriatic Sea and the Strait of Sicily (Fanelli et al., 2018; Riccioni et al., 2018). These scientific approaches could extrapolate new essential information about trophic position of this species, clarifying the organization and composition of trophic networks in demersal environments and the ecological relationships between mesopelagic and epipelagic strata. This knowledge base is fundamental to improve management actions for the preservation of the entire ecosystem, including relevant ecological and commercial fish communities and stocks. Indeed, deepen the knowledge about predators’ trophic relationships and feeding habits is a way to understand the ecological network which sustains overexploited stock and communities, adding new useful information to improve ecosystems conservation and stocks management.

## CONFLICT OF INTEREST

The authors have no conflicts of interest to declare.

## AUTHOR CONTRIBUTIONS

**Claudio D'Iglio:** Conceptualization (lead); Data curation (equal); Investigation (equal); Methodology (equal); Supervision (equal); Validation (equal); Writing – original draft (lead); Writing – review & editing (lead). **Nunziatina Porcino:** Conceptualization (equal); Data curation (lead); Formal analysis (equal); Investigation (lead); Methodology (equal); Software (lead); Supervision (equal); Validation (equal); Writing – original draft (equal); Writing – review & editing (equal). **Serena Savoca:** Data curation (equal); Formal analysis (equal); Writing – review & editing (equal). **Anna Perdichizzi:** Data curation (equal); Funding acquisition (supporting); Investigation (equal); Methodology (equal); Project administration (supporting); Resources (lead). **Adriana Profeta:** Formal analysis (supporting); Investigation (supporting); Methodology (supporting); Project administration (supporting); Resources (supporting); Validation (supporting). **Enrico Armeli Minicante:** Data curation (equal); Investigation (equal); Methodology; Resources (supporting); Supervision (equal). **Davide Salvati:** Data curation (supporting); Investigation (supporting); Methodology (supporting); Resources (supporting). **Francesco Soraci:** Investigation (supporting); Methodology (supporting). **Paola Rinelli:** Conceptualization (equal); Funding acquisition (lead); Methodology (equal); Project administration (lead); Resources (lead); Supervision (lead); Validation (equal); Visualization (supporting); Writing – review & editing (equal). **Daniela Giordano:** Conceptualization (lead); Data curation (equal); Formal analysis (equal); Funding acquisition (equal); Investigation (lead); Methodology (equal); Project administration (equal); Resources (equal); Software (equal); Supervision (lead); Validation (lead); Visualization (lead); Writing – original draft (supporting); Writing – review & editing (supporting).

## Data Availability

All the data are property of U.E. https://datacollection.jrc.ec.europa.eu; https://dcf‐italia.cnr.it/web/#/pages/home
